# BatchFLEX: feature-level equalization of X-batch

**DOI:** 10.1093/bioinformatics/btae587

**Published:** 2024-10-03

**Authors:** Joshua T Davis, Alyssa N Obermayer, Alex C Soupir, Rebecca S Hesterberg, Thac Duong, Ching-Yao Yang, Ken Phong Dao, Brandon J Manley, G Daniel Grass, Dorina Avram, Paulo C Rodriguez, Brooke L Fridley, Xiaoqing Yu, Mingxiang Teng, Xuefeng Wang, Timothy I Shaw

**Affiliations:** Department of Biostatistics and Bioinformatics, H. Lee Moffitt Cancer Center and Research Institute, Tampa, FL 33612, United States; Department of Biostatistics and Bioinformatics, H. Lee Moffitt Cancer Center and Research Institute, Tampa, FL 33612, United States; Department of Biostatistics and Bioinformatics, H. Lee Moffitt Cancer Center and Research Institute, Tampa, FL 33612, United States; Department of Tumor Microenvironment and Metastasis, H. Lee Moffitt Cancer Center and Research Institute, Tampa, FL 33612, United States; Department of Biostatistics and Bioinformatics, H. Lee Moffitt Cancer Center and Research Institute, Tampa, FL 33612, United States; Department of Biostatistics and Bioinformatics, H. Lee Moffitt Cancer Center and Research Institute, Tampa, FL 33612, United States; Department of Malignant Hematology, H. Lee Moffitt Cancer Center and Research Institute, Tampa, FL 33612, United States; Department of Genitourinary Oncology, H. Lee Moffitt Cancer Center and Research Institute, Tampa, FL 33612, United States; Department of Radiation Oncology, H. Lee Moffitt Cancer Center and Research Institute, Tampa, FL 33612, United States; Department of Immunology, H. Lee Moffitt Cancer Center and Research Institute, Tampa, FL 33612, United States; Department of Immunology, H. Lee Moffitt Cancer Center and Research Institute, Tampa, FL 33612, United States; Department of Biostatistics and Bioinformatics, H. Lee Moffitt Cancer Center and Research Institute, Tampa, FL 33612, United States; Department of Malignant Hematology, Children’s Mercy, Kansas City, MO 64108, United States; Department of Biostatistics and Bioinformatics, H. Lee Moffitt Cancer Center and Research Institute, Tampa, FL 33612, United States; Department of Biostatistics and Bioinformatics, H. Lee Moffitt Cancer Center and Research Institute, Tampa, FL 33612, United States; Department of Biostatistics and Bioinformatics, H. Lee Moffitt Cancer Center and Research Institute, Tampa, FL 33612, United States; Department of Biostatistics and Bioinformatics, H. Lee Moffitt Cancer Center and Research Institute, Tampa, FL 33612, United States

## Abstract

**Motivation:**

Integrative analysis of heterogeneous expression data remains challenging due to variations in platform, RNA quality, sample processing, and other unknown technical effects. Selecting the approach for removing unwanted batch effects can be a time-consuming and tedious process, especially for more biologically focused investigators.

**Results:**

Here, we present BatchFLEX, a Shiny app that can facilitate visualization and correction of batch effects using several established methods. BatchFLEX can visualize the variance contribution of a factor before and after correction. As an example, we have analyzed ImmGen microarray data and enhanced its expression signals that distinguishes each immune cell type. Moreover, our analysis revealed the impact of the batch correction in altering the gene expression rank and single-sample GSEA pathway scores in immune cell types, highlighting the importance of real-time assessment of the batch correction for optimal downstream analysis.

**Availability and implementation:**

Our tool is available through Github https://github.com/shawlab-moffitt/BATCH-FLEX-ShinyApp with an online example on Shiny.io https://shawlab-moffitt.shinyapps.io/batch_flex/.

## 1 Introduction

Studying the reprogramming of the immune system in cancer cells often requires in-depth integration of omics-generated data, including microarray, RNA sequencing, and mass spectrometry. However, a common issue when combining different batches of datasets is to resolve the technical variation contributed during generation, including processing protocols, platform vendors, and personnel. Each technical variable can introduce an unwanted batch effect and confounding factors (from both observable and unobservable variables) that influence downstream analyses. Ultimately, batch effects can obscure the biological variable and condition of interest. Incorrectly addressing these confounders in the data can lead to erroneous conclusions, especially when integrating the molecular profiling of immune cells and cancer patient data. Batch correction methods can be separated into two major categories: (i) location–scale (L/S) adjustment methods and (ii) matrix factorization methods. Location-scale adjustment methods adjust the mean or variance of the dataset, such as mean-centering (Nygaard *et al.* 2016), Combat (Johnson *et al.* 2007), and LIMMA (Ritchie *et al.* 2015). Matrix factorization methods estimate the underlying batch factor to correct, such as HARMAN (Oytam *et al.* 2016), RUVg (Risso *et al.* 2014), and SVA (Leek and Storey 2007). Each method has its unique advantages tailored to the complexity of the dataset. But, knowing when to select a specific batch correction method can be difficult. While several publicly available servers can perform batch correction, such as ExploBATCH (Nyamundanda *et al.* 2017), BATCHQC (Manimaran *et al.* 2016), and BATCHserver (Zhu *et al.* 2021), these web resources are generally limited to a single correction and diagnosis strategy. Here, we present BatchFLEX, a correction-agnostic shiny app that simplifies the batch correction procedure by centralizing and streamlining commonly applied methods (see Table 1 for list of functions). BatchFLEX provides several methods of evaluating the corrected matrix, including dimension reduction, clustering evaluation, batch identification, latent variable analysis, and principal variance component analysis (Li *et al.* 2009). BatchFLEX streamlines and simplifies the batch correction process to ensure a consistently effective batch correction, while providing real-time comparison of altered gene expression, deconvoluted immune markers, and pathways. The BatchFLEX app requires no coding experience and can easily be updated with additional batch correction methods as they become available. BatchFLEX is available through GitHub, shiny.io, and docker, and a companion R function for a single access point to all the batch correction methods and evaluation strategies.

**Table 1.** Comparisons of features.^a^

	BatchFLEX	ExploBATCH	BATCH QC	BATCH Server
Batch correction methods				
Combat	X	X	X	X
Limma	X			
CombatSeq	X		^b^	
Mean centering	X			
Harman	X			
RUVg	X			
correctBatch		X		
Surrogate variable analysis	X		X	
Analysis of batch effects				
Principal component analysis	X	X	X	
Scree plot	X	X		
Visualization of clusters	X		X	
Elbow analysis	X			
Silhouette analysis	X			
Dunn index evaluation	X			
Variance dist. analysis	X		X	
Relative log expression	X		X	
Individual gene analysis	X			
Surrogate variable analysis	X		X	
PVCA	X		^b^	X
UMAP	X			X
Heatmap	X		^b^	
Additional features				
Outlier detection	X		X	
Export corrected matrix	X	X	^b^	X
Export plots and figures	X	X	X	X
Companion R package	X		X	

a PVCA: Principal Variance Component Analysis. UMAP: Uniform Manifold Approximation and Projection. Dist: Distribution.

b Features absent in the original publication but reported on the GitHub page (Accessed 8/1/2024).

## 2 Features

BatchFLEX is a Shiny App that can be accessed on a web server or installed locally in R or via a docker container. BatchFLEX is designed to guide the user through four key steps.

1. Input of a data matrix and metafile.
2. Assessment of the batch effect.
3. Removal of the batch effect.
4. Side-by-side comparisons to evaluate each correction method

Finally, the user can download the batch correction and summary statistics assessing the impact of the batch correction method, with downstream functionality to assess the impact of the batch correction on pathways and immune deconvolution algorithms.

### 2.1 Required user input

**Figure 1.**
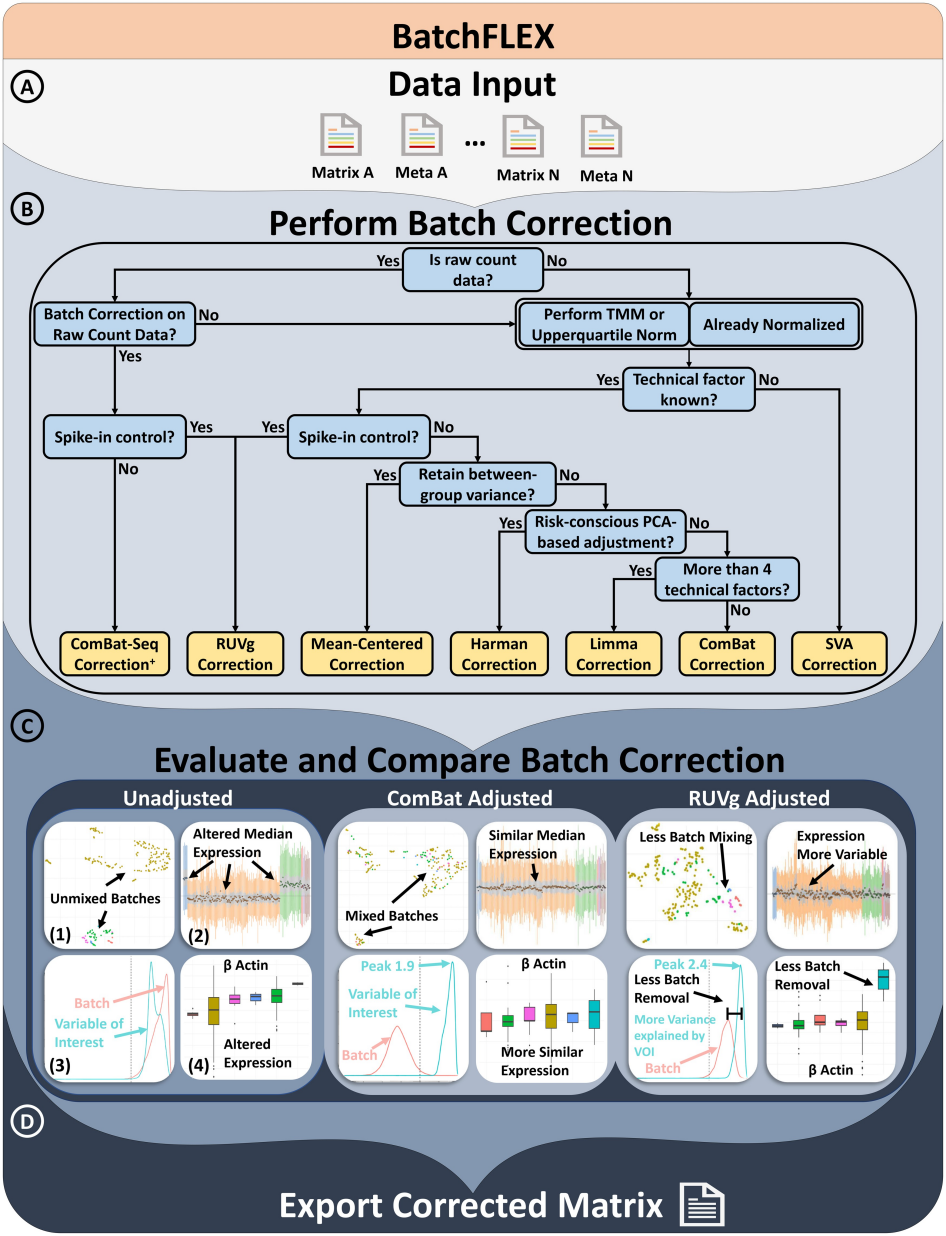
Overview of the BatchFLEX analysis tool workflow. (A) Upload a merged matrix and meta file containing multiple studies. Use MergeQC app if needed. (B) Perform batch correction using one of the app's methods. A decision tree is provided to assists in guiding method selection. (C) Evaluate the batch correction efficacy using side-by-side comparison plots with examples from ComBAT and RUVg. (1) UMAP plot: Check if clustering is driven by the batch effect; ideally, data should be intermixed and driven by the variable of interest. (2) Relative log expression plot: Assess batch effect impact on median expression; broad expression should be similar across datasets. (3) Explanatory variables plot: Estimates the variance explained by the annotated feature; the variable of interest should explain more variance than the batch effect. (4) Boxplot: Can evaluate expression of gene and features, such as housekeeping genes. (D) Export the corrected matrix for downstream analysis. + indicating the feature will only be present in the app when count-based matrix is provided as input

BatchFLEX requires two input files. (i) An expression matrix based on human or mouse gene symbols, and (ii) a metafile with sample names (matching the expression matrix) in the first column with batch information and sample annotation in the next series of columns (Fig. 1A). The optimal use of the app is to include at least one column with annotated technical factors, which can be leveraged as a batch correction factor. The program can perform additional preprocessing and normalization of the matrix, such as log transformation, quantile normalization, and Trimmed Mean of M-values (TMM) normalization for count-based data. While the app is intended for post-batch correction, BatchFLEX can perform unsupervised and supervised evaluations of the batch effect on the uncorrected matrix (see Section 2.3).

### 2.2 Batch correction methods

BatchFLEX includes a wide variety of well-documented and commonly used batch correction methods such as Limma (Ritchie *et al.* 2015), ComBat (Johnson *et al.* 2007), ComBatSeq (Zhang *et al.* 2020), Mean-Centering (Nygaard *et al.* 2016), Harman (Oytam *et al.* 2016), and RUVg (Risso *et al.* 2014). Batch correction can easily be applied to the input matrix in real-time based on the user-selected factor and covariates, and the batch-corrected matrix can then be compared side-by-side. To facilitate the selection of the batch correction strategy, a simplified decision tree is provided to guide users in maximizing the advantages of each correction algorithm (Fig. 1B). These factors include (i) Is the input data based on raw count or normalized based on a scale factor? (ii) Are the technical factors known to be key drivers of the variance? (iii) Is there an expression control, such as ERCC spike-in or housekeeping genes? (iv) Is there a primary interest in retaining gene-level variance? (v) Is there a preference for leveraging PCA as a mode for correction? (vi) Are there more than four technical factors influencing the batch? As an example of the post-corrected difference, we compare the result from Combat versus RUVg (Fig. 1C). Our results highlight Combat was better able to correct the batch effect but does not preserve the cross-sample variability as well as RUVg. Altogether, our tool aims to be flexible and adaptable to diverse data types while addressing most use cases and scenarios.

### 2.3 Batch evaluation strategies

BatchFLEX provides four significant methods of batch evaluation, which can be categorized into (i) unsupervised analysis, (ii) variance analysis of known batch effects, (iii) estimating unknown batch effects, and (iv) comparative expression analysis. **Unsupervised analysis** includes dimension reduction into low dimensions and clustering. The data can be visualized in principal components via PCA, which can be annotated by assigning color to any factor of interest. BatchFLEX also generates a table displaying the contribution of variance by each factor. BatchFLEX performs cluster analysis of k-means generated clusters using Elbow, Silhouette, and Dunn plots. These analyses can help users determine the optimal number of clusters and whether the clusters correlate well with the biological variable of interest. The cluster’s association with the batch effect can be evaluated through diversity measures of heterogeneity and evenness. Next, **the variance analysis of known batch effects** examines the variation contributed by annotated technical factors, such as study type, platform, and sequencing type. BatchFLEX provides two major functions for this assessment: relative log expression plots and explanatory variable plots. Users can organize the data according to known technical factors and visualize unwanted variation from the batch effect using the sample-wise relative log expression plot. The explanatory variables and density analysis are included to assess whether each gene is associated more with the batch effect or the variable of interest by displaying the distribution of R-squared values across all genes for each user-selected variable. **Batch estimation** is performed with surrogate variable analysis to identify hidden batch effects by inferring latent variables in orthogonal space (Leek and Storey 2007). BatchFLEX then provides the posterior probability that each gene is associated with the latent variable implemented in the SVA package. Additionally, BatchFLEX can determine the impact of a batch effect or biological variable of interest at an individual gene level using a boxplot and using statistical measures of significance from the Wilcoxon rank-sum test, the *t*-test, the Kruskal Wallis test, or ANOVA. The boxplot and RLE plot also allow users to assess if outliers are present at a sample level or a gene level. Each of these methods of visualization and statistical analysis helps the user assess if the batch effect is significant and if correction is necessary. Moreover, BatchFLEX provides real-time immune deconvolution by ImmDeconv (Sturm *et al.* 2020) and single-sample Pathway analysis as implemented in GSVA (Hanzelmann *et al.* 2013). Each analysis is rendered side-by-side for easy comparison. Users can easily switch between batch correction methods, allowing on-the-fly comparisons of batch correction methods to ensure that the most optimal method is chosen for a particular dataset. The output can easily export the updated matrix for downstream analysis and any desired diagnostic can then be downloaded as a single ZIP file (Fig. 1D).

## 3 Tutorial and example data

### 3.1 Tutorial

The Supplementary Materials and package vignettes include detailed implementation, tutorial, and file input requirements. The package vignettes also include a function for generating simulated data. We have also provided a video tutorial page with step-by-step instruction on how to navigate the user interface (Supplementary Fig. S2). Briefly, these steps include:

1. Installation
2. Load Example Data
3. Input User Data
4. Assessment of Batch Effect
5. Batch Correction
6. Evaluating the Batch Correction
7. Editing figure parameters
8. On-the-fly Comparisons
9. File Export

### 3.2 Example data

As an example, we analyzed six microarray profiles of immune cells derived from the ImmGen Repository (Heng *et al.* 2008). The ImmGen microarray data consists of murine immune lineages of T cell, B cell, Myeloid cells, stromal cells, and early precursor/progenitor populations of leukocytes from GSE112876, GSE15907, GSE37448, GSE60336, GSE60337, and GSE75202 (Heng *et al.* 2008, Desch *et al.* 2011, Painter *et al.* 2011, Malhotra *et al.* 2012, Elpek *et al.* 2014, Mostafavi *et al.* 2016). First, we performed log-normalization followed by quantile normalization, which can reduce the level of variability across different studies. Then, we performed batch correction on the effect of the study using Combat under a parametric empirical Bayes framework. We then performed dimension reduction of the data via Uniform Manifold Approximation and Projection (UMAP). The data was denoised by PCA and the umap function, implemented in the “umap” R library, was executed with a min.distance of 0.1, N Neighbors of 15, and using “Pearson” (Supplementary Fig. S3A). Our data showed that prior to batch correction, the study batch effect is the primary driver of the variance in the UMAP projection with the immune cell type diffused throughout the projection (Supplementary Fig. S3B). And following the batch correction, the individual cell types are more closely clustered in the UMAP space (Supplementary Fig. S3C). Next, we examined Myb, a transcription factor that is associated with development in lymphocytes, which was found altered in expression rank in immune cell types before-and-after batch correction (Supplementary Fig. S3B and C). Prior to correction Myb was most highly expressed in Mast cells, and after correction Myb was ranked more highly expressed in precursor T and B cells. The analysis indicates that the expression of certain lineage specific transcription factors might be impacted by the batch correction strategy. To provide an example of batch correction on pathway scores, we compared the summarized expression of chemokines based on the chemokine-12 gene signature (Messina *et al.* 2012), consisting of CCL2, CCL3, CCL4, CCL5, CCL8, CCL18, CCL19, CCL21, CXCL9, CXCL10, CXCL11, and CXCL13 (Supplementary Fig. S3B, and C). The batch correction had a significant impact on the chemokine expression levels in innate lymphocytes, monocytes, and granulocyte cells, revealing a global pattern of chemokines being most highly expressed in stroma and myeloid cells, which are more likely to express these cytokine attractants to recruit immune cells, such as lymphocytes. Altogether, we highlight the impact of the batch correction in altering gene expression and gene signatures across cell type, which enables an improved interpretation of these expression markers in the ImmGen dataset.

## 4 Conclusion

BatchFLEX provides the most comprehensive batch correction and evaluation strategy to maintain the biological variable of interest. BatchFLEX is designed to streamline the batch correction strategy, while offering real-time diagnosis and analysis of the post-corrected data. The tool can be accessed through the web or set up locally on your personal computer at (https://shawlab-moffitt.shinyapps.io/batch_flex/) or (https://github.com/shawlab-moffitt/BATCH-FLEX-ShinyApp). While our method is designed for a broad general audience, a companion BatchFLEX R package (https://github.com/shawlab-moffitt/BATCHFLEX) is designed for advanced users to streamline their data analysis with access to additional functions for batch correction and diagnosis.

## Supplementary Material

btae587_Supplementary_Data

## Data Availability

BatchFLEX is available through Github https://github.com/shawlab-moffitt/BATCH-FLEX-ShinyApp with an online example on Shiny.io https://shawlab-moffitt.shinyapps.io/batch_flex/.
