# Supplementary material for: BatchFLEX: feature-level equalization of X-batch
Source: Bioinformatics. 2024 Oct 3;40(10):btae587. doi: 10.1093/bioinformatics/btae587 (PMC11486499; doi:10.1093/bioinformatics/btae587)
Supplement: btae587_Supplementary_Data [file btae587_supplementary_data.docx]

**Supplementary Material: Figures and Tutorial**

BatchFLEX: Comprehensive software for assessing and correcting batch effects, comparing batch correction methods, and exporting corrected matrices for downstream analysis

**Introduction**

BatchFLEX is an intuitive Shiny App that can be viewed as a web interface through shiny.io or accessed locally by downloading and installing an app.R or docker container, or by installing an R-package. BatchFLEX is designed with a clean user interface and easy to follow steps to prompt users to input a data matrix and meta file, to select and implement the desired method of batch correction, to assess the impact of the batch correction method using side-by-side comparisons of pre and post corrected graphs and statistics, and finally to export a corrected matrix and accompanying diagnostics for downstream analysis. The intuitive user interface allows for hands-on evaluation and selection of the most optimal batch correction method for any dataset and for users with any background. BatchFLEX is the most comprehensive batch correction and evaluation tool and can easily be implemented into any pipeline using the R-package wrapper. Notable features of BatchFLEX include implementation of a wide variety of batch correction methods such as ComBat, ComBatSeq, Harman, LIMMA, RUVg, and Mean centering, incorporation of multiple different types of evaluation methods such as PCA, cluster analysis, gene-variable association analysis, diversity analysis, scree plots, SVA, UMAP, RLE, boxplots, and heatmaps, and inclusion of a simple and comprehensive export feature for use of corrected matrices in downstream analysis and for record keeping to justify the correction method selection for publications.

An overview of the BatchFLEX suite of tools can be found on our GitHub page (<https://github.com/shawlab-moffitt/BATCH-FLEX-ShinyApp>), which includes source code, example data, and an installation guide.

Additionally, BatchFLEX can be access through shiny.io at (<https://shawlab-moffitt.shinyapps.io/batch_flex/>).

A dockerized version of the app can be found at (<https://github.com/shawlab-moffitt/BATCH-FLEX-ShinyApp/tree/main/ShinyDocker>).

**Installation**

The BatchFLEX suite can be downloaded (cloned) and installed through the GitHub repository. The downloaded file can be unzipped to a destination folder, which should be set as the working directory or file path. Of note, some of the example files (e.g., gene set files) use relative paths, so the program may fail to identify the file if a working directory is not properly set.

- Install BatchFLEX suite GitHub repository
  - git clone (<https://github.com/shawlab-moffitt/BATCH-FLEX-ShinyApp>)
  - Download and unzip repository
- Set working directory to BatchFLEX folder
- Install required R packages
  - Suite of tools was built on R version 4.2
  - R script for package installation is provided in the “1-Getting_started” folder
- To Run the App, simply click on the app.R file and click on the Run App button in RStudio

**Easy Start**

BatchFLEX provides a homepage to guide users to the appropriate tabs to initiate their analysis. To begin the analysis, users can click on the **“Get Started”** button, which will navigate the user to the data input panel of BatchFLEX. The **“Need Tutorial?”** button will navigate users to the tutorial tab for additional guidance (**Supplementary Figure S1A**). The homepage also provides a link to the BatchFLEX shiny GitHub, a link to the GitHub of our companion R package, and the ability to open a separate instance of the BatchFLEX shiny app (**Supplementary Figure S1B**). To help users merge several datasets, MergeQC can be accessed from the homepage or downloaded to the user’s local environment from the GitHub page (**Supplementary Figure S1C**).

**
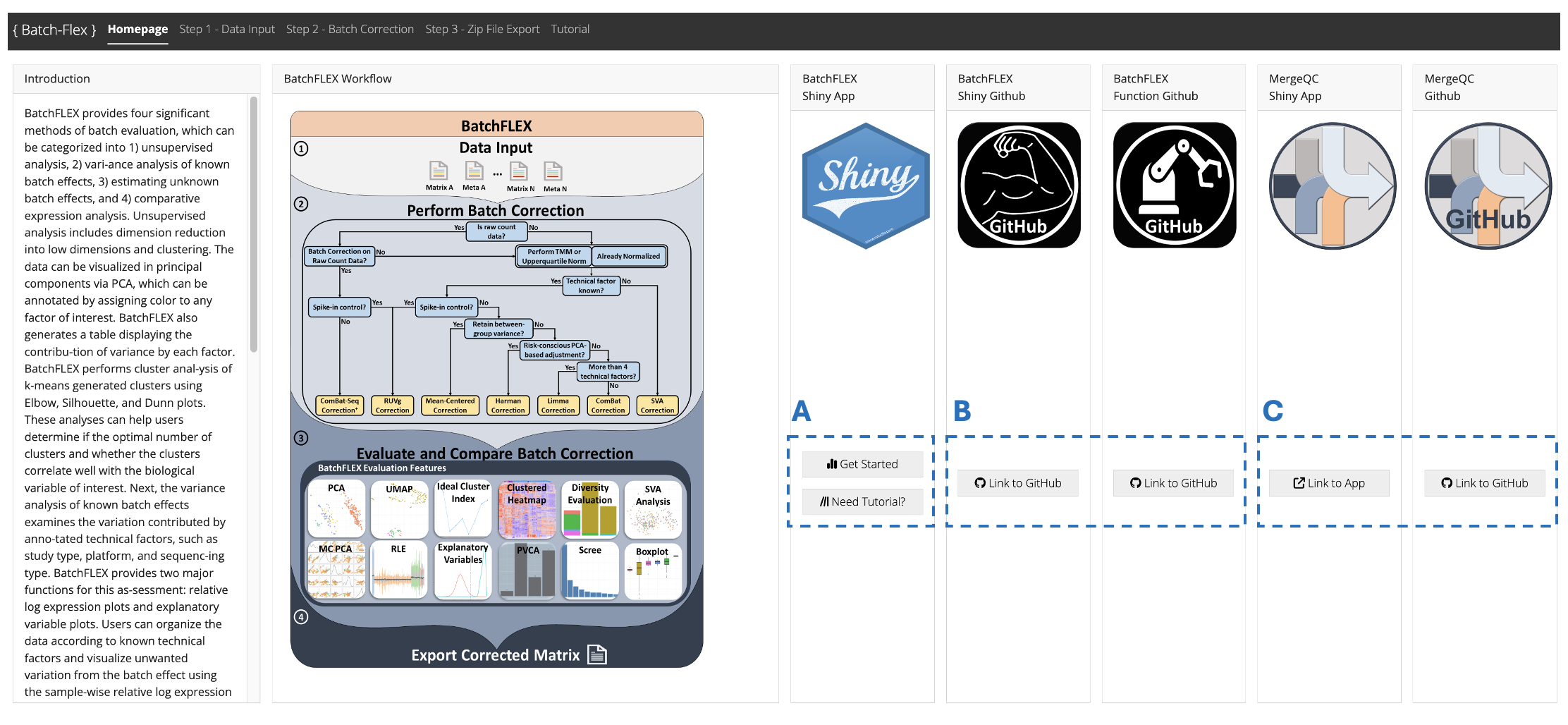
**

**Supplementary Figure S1.** BatchFLEX Homepage. **A)** Users can click on “Get Started” to initiate analysis and can click on “Need Tutorial” to access the tutorial page. **B)** Links to the GitHub page. **C)** Link to the MergeQC Shiny app and GitHub page.

**Video Tutorial**

We have integrated a thorough tutorial within BatchFLEX to help users quickly and easily understand how to use our app (**Supplementary Figure S2**). To access the tutorial, users can simply click the **“Need Help?”** button from the homepage. Alternatively, the user can click on the “**Tutorial**” tab as part of the top navigation bar. On the tutorial page, the left panel provides a list of major functions in BatchFLEX, which the user can click to review the tutorial content. Each tutorial contains a video example of the function (middle panel) with information to guide the user through the batch correction process (right panel).  **
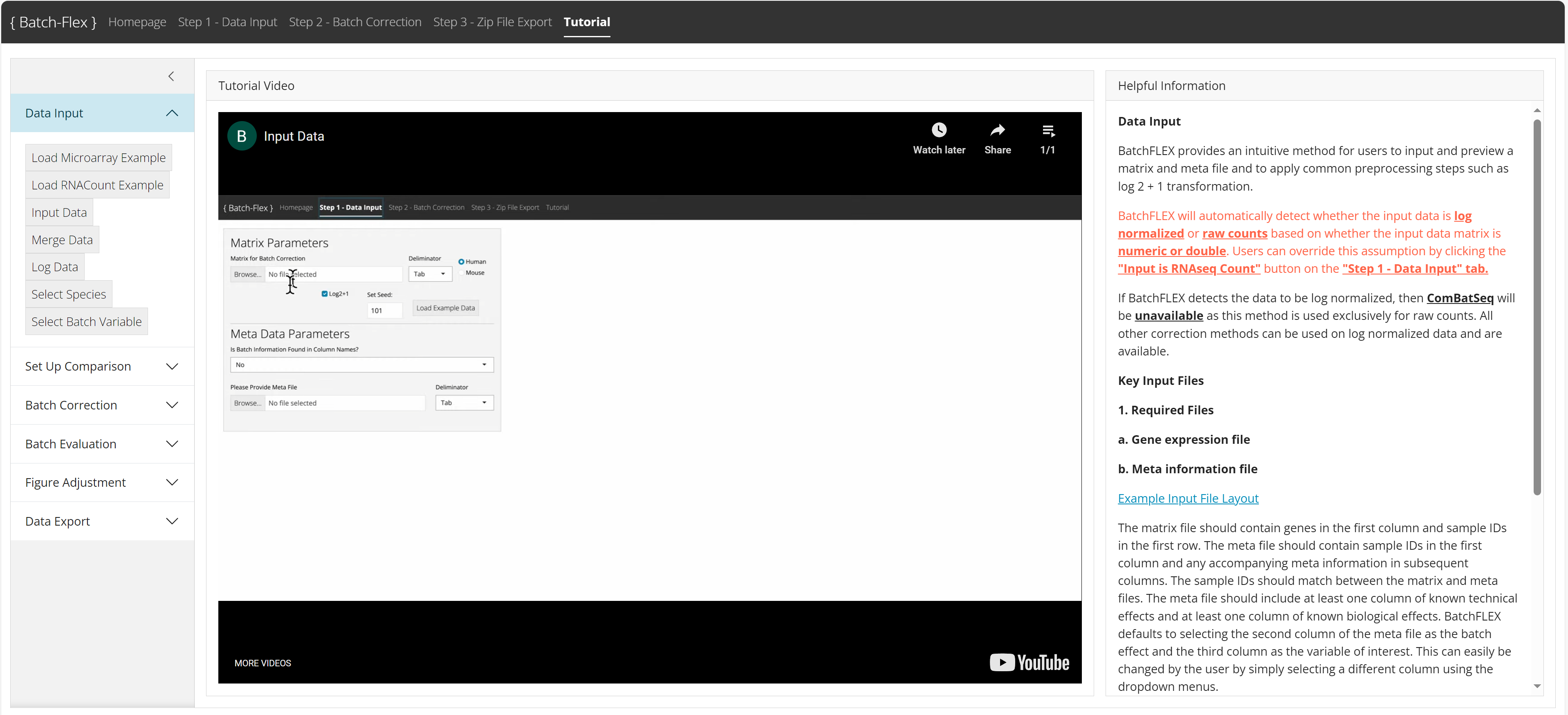
**

**Supplementary Figure S2.** Video tutorial page. A list of topics is shown in the left panel. Video tutorial of the function is shown in the middle panel. Helpful information about the function is shown in the right panel.

**IMMGEN Example**


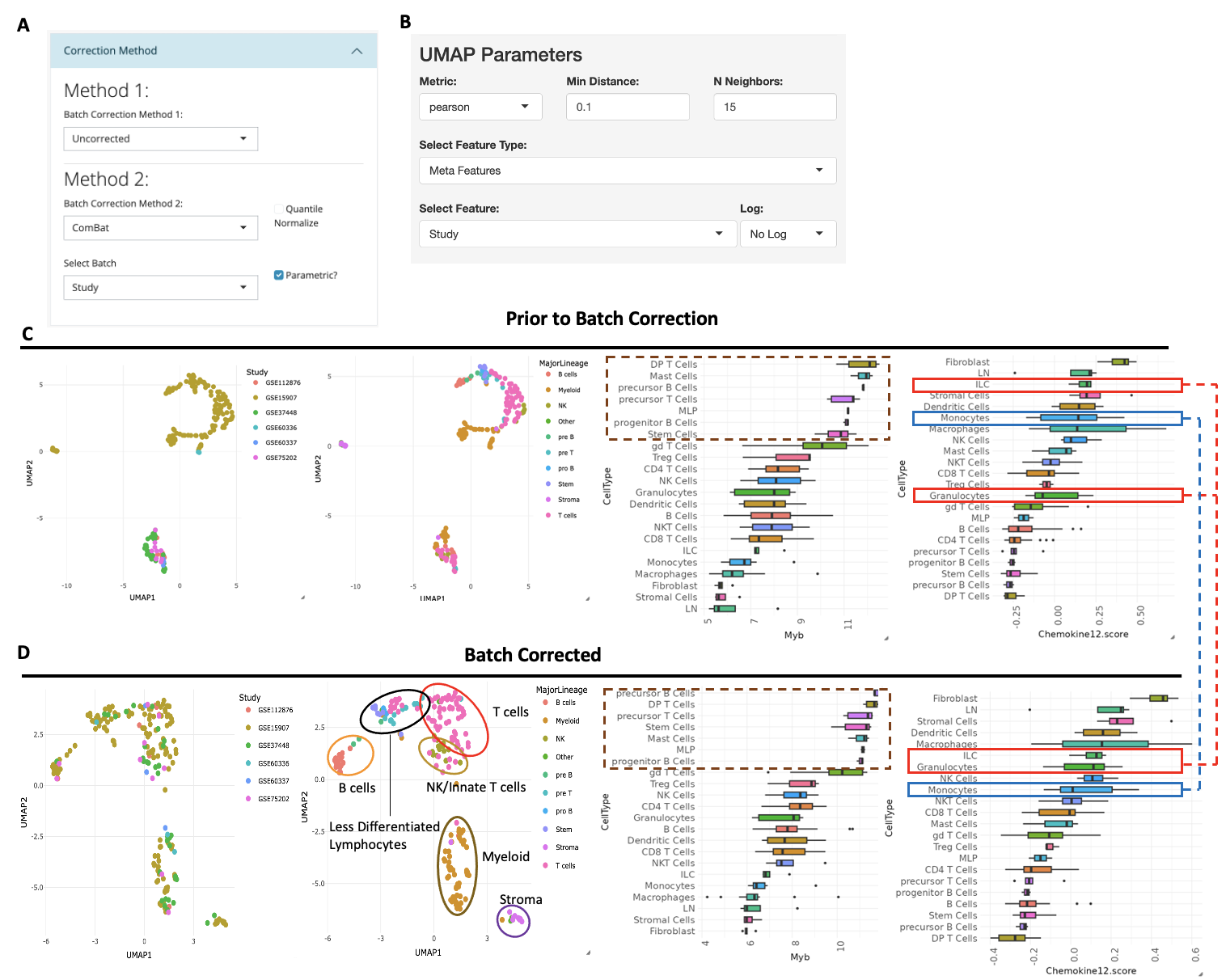


**Supplementary Figure S3.** ImmGen batch correction example. Screen shot of the batch correction method and parameters **(A)** and UMAP parameters **(B).** The uncorrected data **(C)** and the batch corrected data **(D)** are presented as a UMAP projection, Myb expression, and Chemokine 12 pathway scores to highlight differences before and after correction.

**Accompanying R Package**

For users with some coding experience, an accompanying R package has been developed with most of the same functionality as the BatchFLEX app. Some advantages of the package include being able to merge datasets and being able to generate simulated data. Additionally, the R package allows users to generate all plots and matrices for all correction methods in a single export folder using only a few lines of code. If a user has multiple datasets to batch correct, BatchFLEX can be ran in a loop in the background. The R package also automatically arranges all plots into a single figure for each correction method. Boxplots are not available strictly in the R package as it is a more in depth analysis method that requires user input, however, the Shiny App can be called within the R function for this type of analysis.

The GitHub repository can be found at: (<https://github.com/shawlab-moffitt/BATCHFLEX>)

A package tutorial can be found at: (<https://github.com/shawlab-moffitt/BATCHFLEX/tree/main/vignettes>)
